# Supplementary figures and images for: Systems biology elucidates the distinctive metabolic niche filled by the human gut microbe Eggerthella lenta
Source: PLoS Biol. 2023 May 19;21(5):e3002125. doi: 10.1371/journal.pbio.3002125 (PMC10234575; doi:10.1371/journal.pbio.3002125)

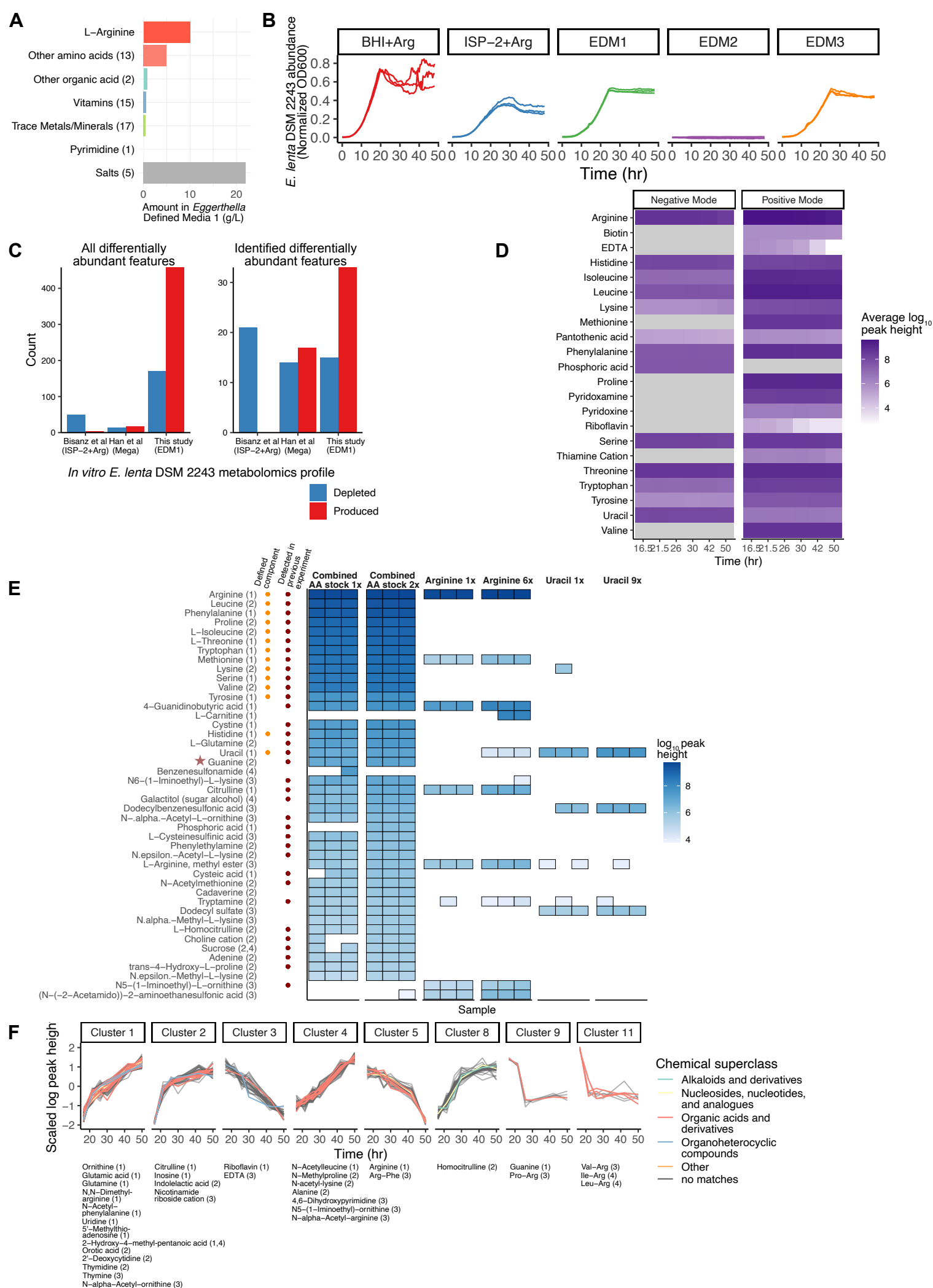

Supplement: S1 Fig — Related to Fig 1. (A) Summary of the composition of EDM1 media. The number in parentheses indicates the number of specific compounds in each category. (B) Growth of E. lenta DSM 2243 in 2 commonly used media conditions (Brain Heart Infusion supplemented with L-arginine, and ISP-2 media supplemented with L-arginine), compared with 3 initial defined media formulations. (C) Comparison of total number of differentially abundant features and identified differentially abundant features in this experiment compared to previous metabolomics profiling of E. lenta. The combination of chemically defined culture media and untargeted metabolomics methods used in this experiment allowed for greater detection of metabolites produced by E. lenta. Produced and depleted features were defined as having an absolute log2 fold change vs. sterile control media greater than 0.5. (D) Metabolomics profiling of compounds known to be present in the chemically defined media formulation EDM1. A total of 22 media compounds were detected, most of which were not significantly depleted in E. lenta cultures over time. (E) Identified compounds detected by untargeted metabolomics in freshly prepared EDM1 media stock solutions. The heatmap shows observed peak heights for features detected from either freshly prepared sterile stock of 14 amino acids plus uracil (“Combined AA stock”), sterile L-arginine solution, or sterile uracil solution. Values are only shown for samples where the intensity was more than 3 times the average value in blank control samples. Guanine is indicated with a star based on its rapid depletion in E. lenta cultures. Its detection in the amino acid solution suggests that it may be a background contaminant from an undetermined amino acid component. The yellow-orange dots indicate compounds that are defined components of the media recipe. The dark red dots denote metabolite features that were previously observed in sterile controls in the time course experiment shown in Fig 1, based o [file pbio.3002125.s001.pdf]

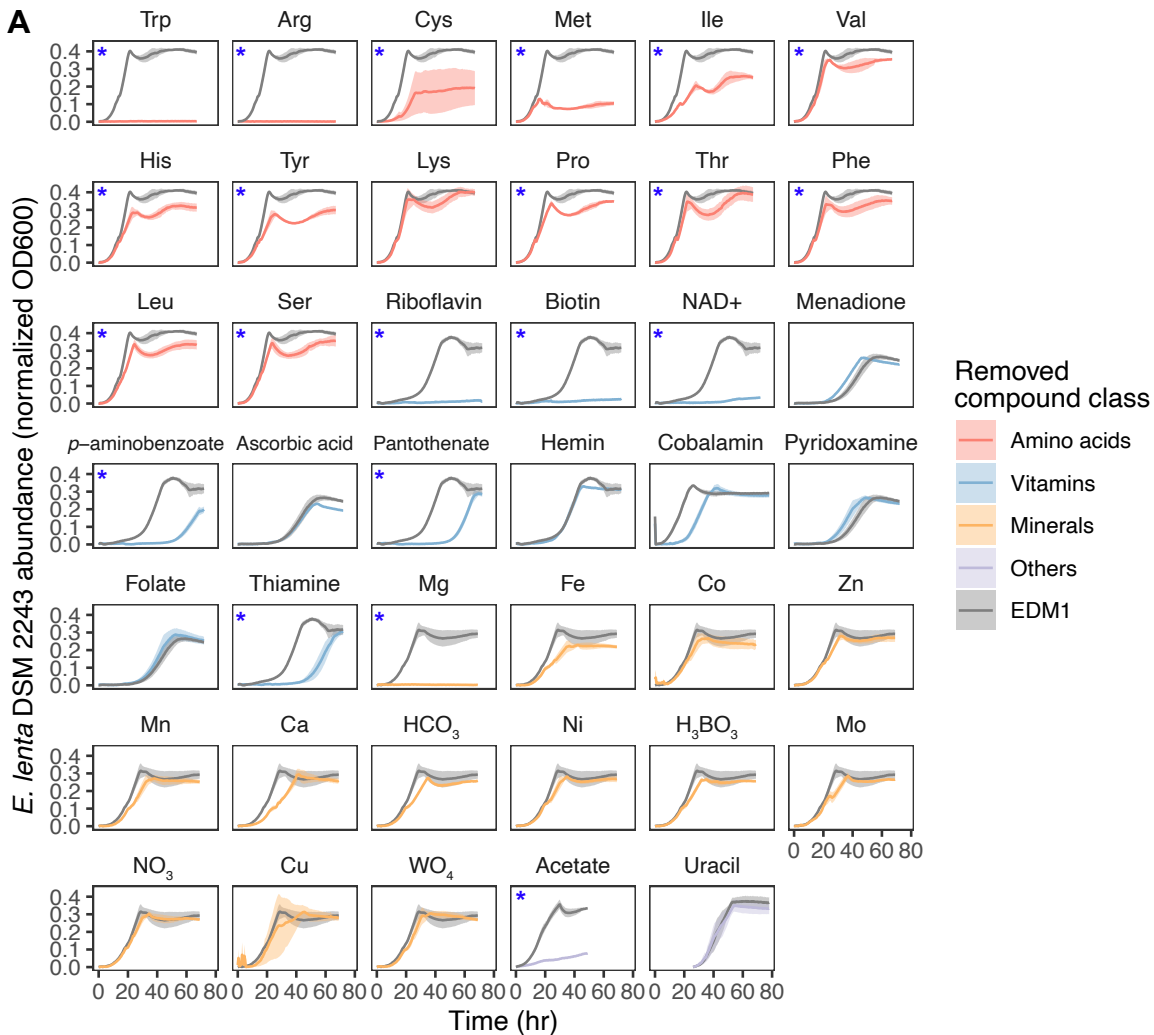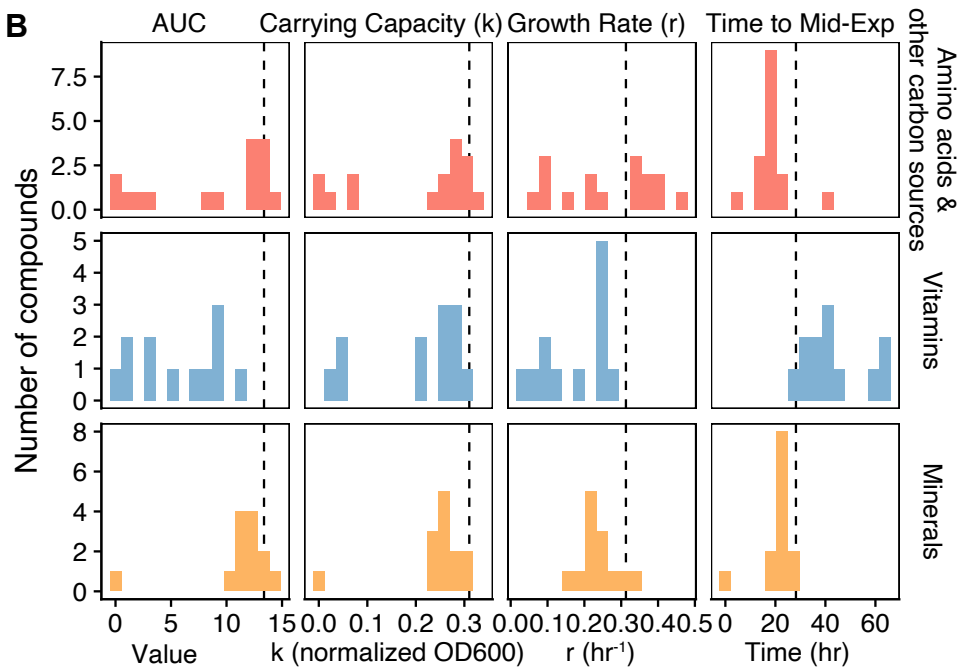

Supplement: S2 Fig — Related to Fig 1. (A) Growth curves for E. lenta DSM2243 growth in EDM1 media with individual media components removed. Gray curves indicate growth in full EDM1 media in the same experiment. Curves are shown as mean ± standard error. A blue asterisk indicates a significance difference in the area under the empirical growth curve with and without the compound (Wilcoxon rank-sum test, FDR-adjusted p < 0.2). (B) Distribution of median effects of removal of all tested compounds on growth parameters estimated by a logistic model. The dotted line indicates the median parameter estimate for the full EDM1 media across all experiments. Parameters were fitted with a logistic model implemented by the R package growthcurver. EDM1, Eggerthella Defined Media 1; FDR, false discovery rate. (PDF) [file pbio.3002125.s002.pdf]

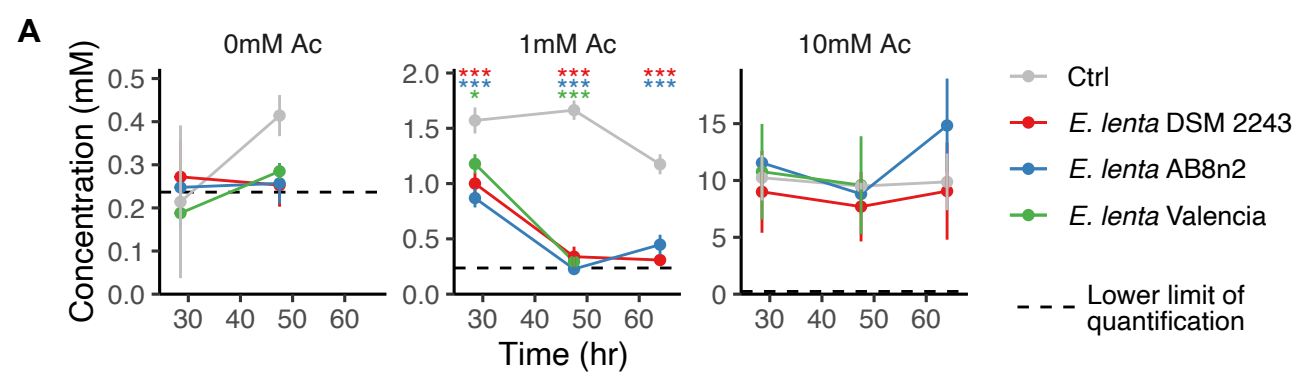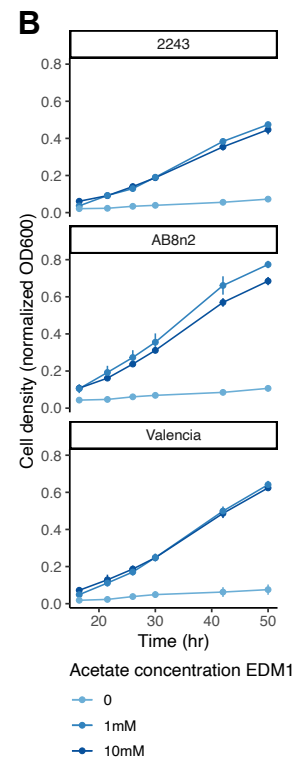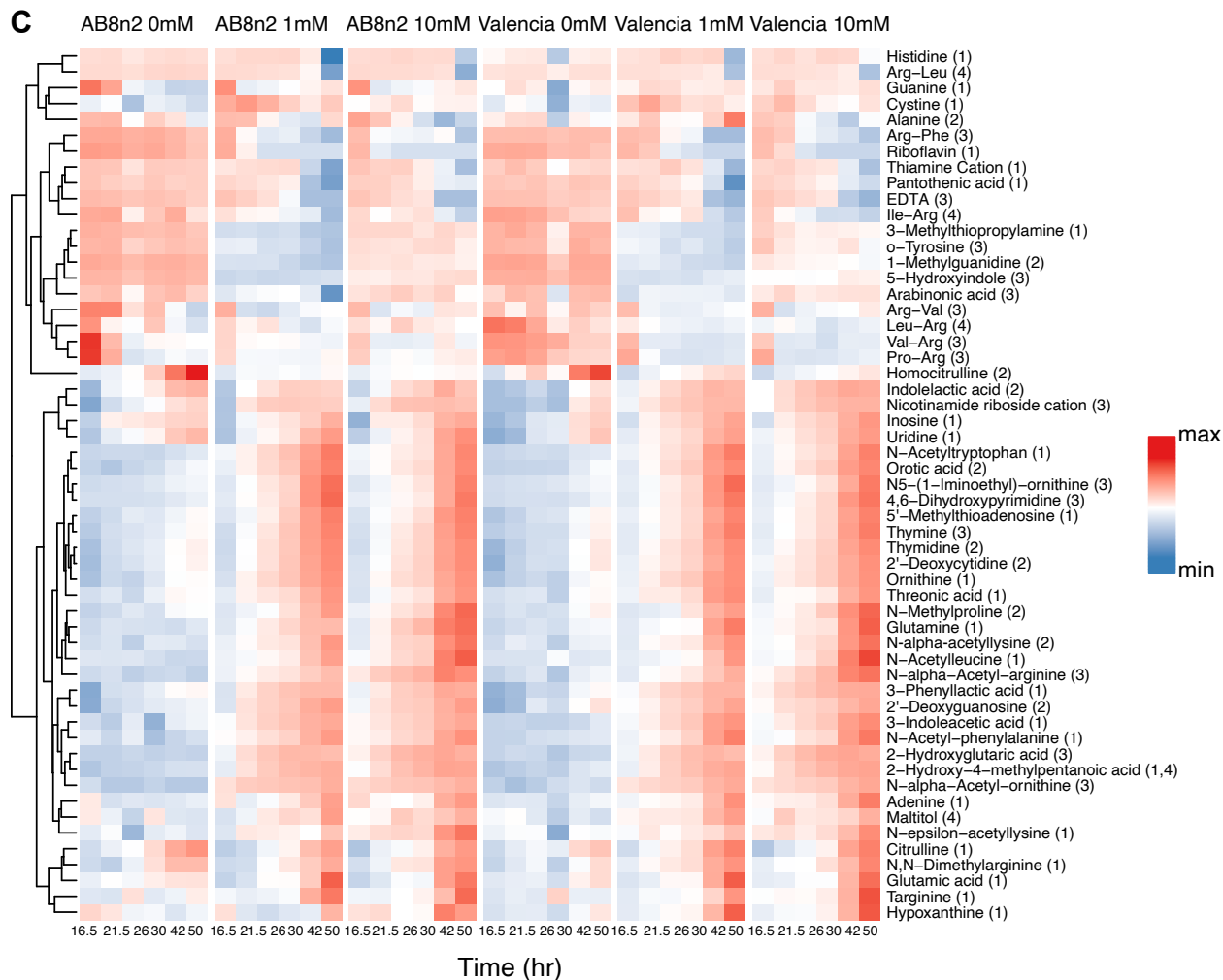

Supplement: S3 Fig — Related to Fig 2. (A) Targeted quantification of acetate depletion in E. lenta EDM1 cultures. Acetate was measured at 2–3 time points in supernatant samples from 3 E. lenta strains during growth in EDM1 as well as sterile controls. Quantification was performed using a method for derivatization of carboxylic acids with 3-nitrophenylhydrazine and N-(3-dimethylaminopropyl)-N′-ethylcarbodiimide followed by targeted LC-MS/MS. Error bars show mean +/− standard error. Linear models of acetate concentration versus strain and time point were inferred for each media group, and differences from controls under the resulting model were estimated using Dunnett’s method. * indicates p < 0.05, *** indicates p < 0.001. (B) Growth of 3 E. lenta strain isolates in EDM1 with 0, 1, or 10 mM sodium acetate. Mean +/− standard error across 3 replicates is shown. (C) Acetate-responsive metabolites in supernatants from E. lenta AB8n2 and E. lenta Valencia. Metabolites shown are those that were assigned an identification, were differentially abundant compared with sterile controls (FDR-adjusted p < 0.2), and had significantly different trajectories over time in the presence vs. absence of acetate in either strain (based on smoothing spline regression with the R package santaR, FDR-adjusted p < 0.25). Values shown are scaled log-transformed peak heights. The number in parentheses indicates the MSI confidence level for each metabolite annotation (see Materials and methods). EDM1, Eggerthella Defined Media 1; FDR, false discovery rate; MSI, Metabolomics Standards Initiative. (PDF) [file pbio.3002125.s003.pdf]

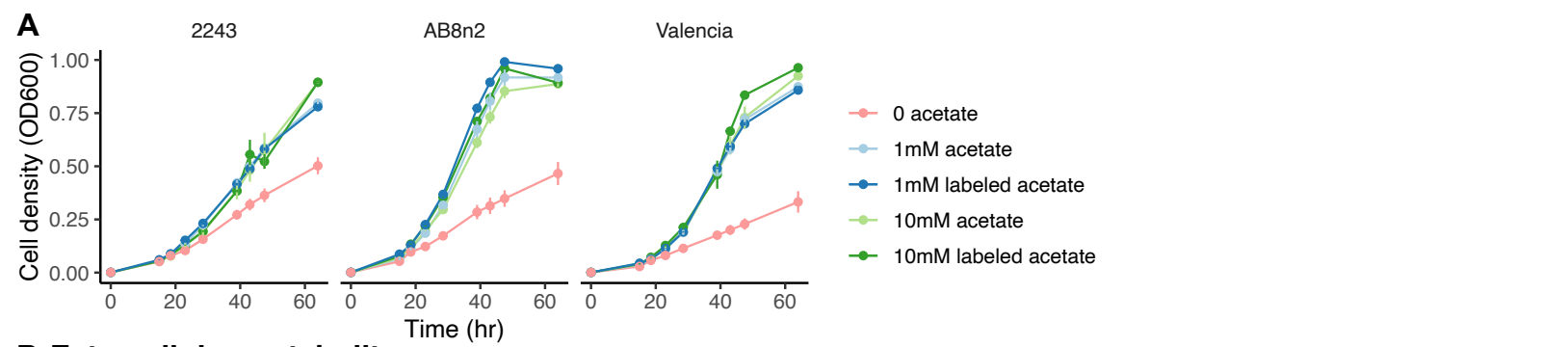

## B Extracellular metabolites

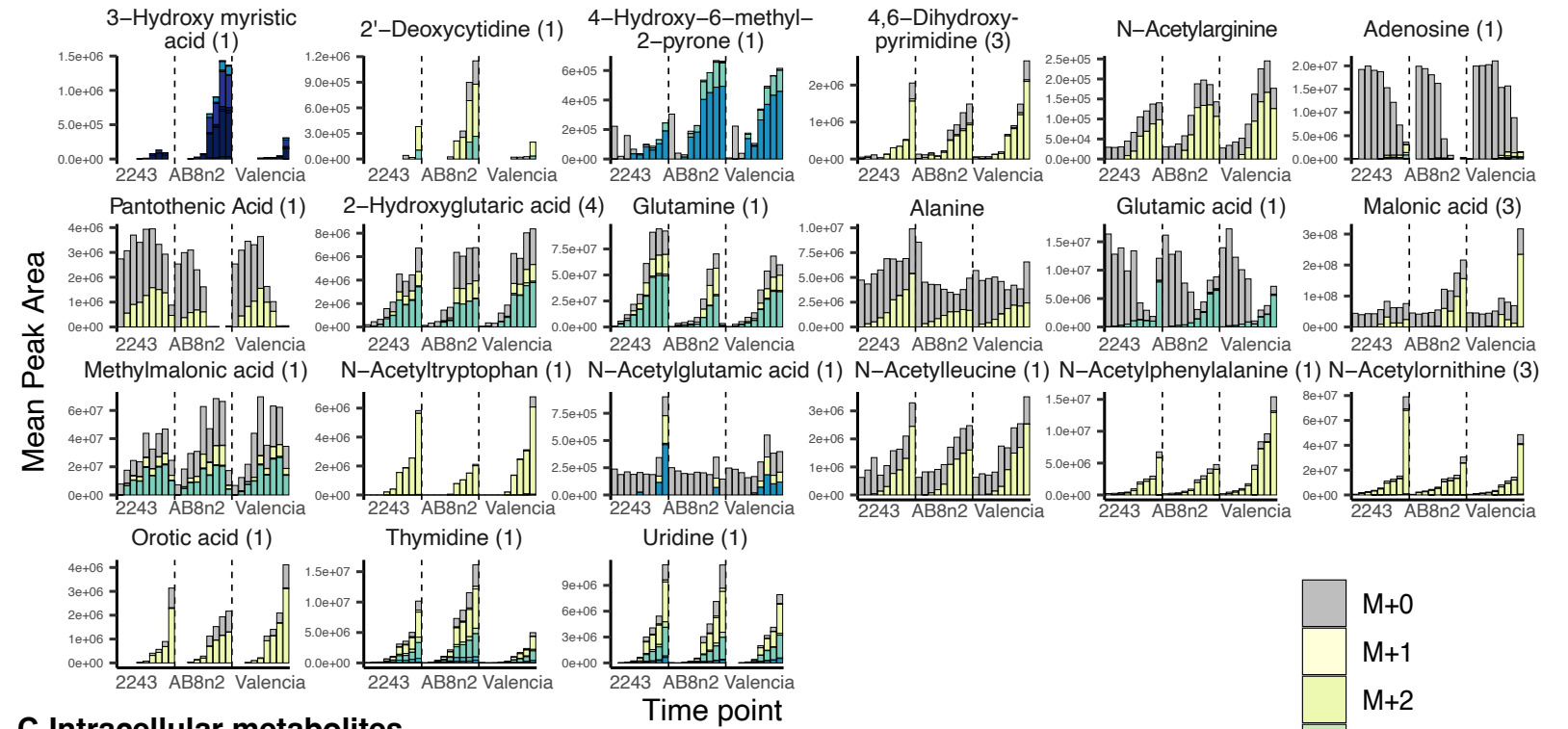

## C Intracellular metabolites

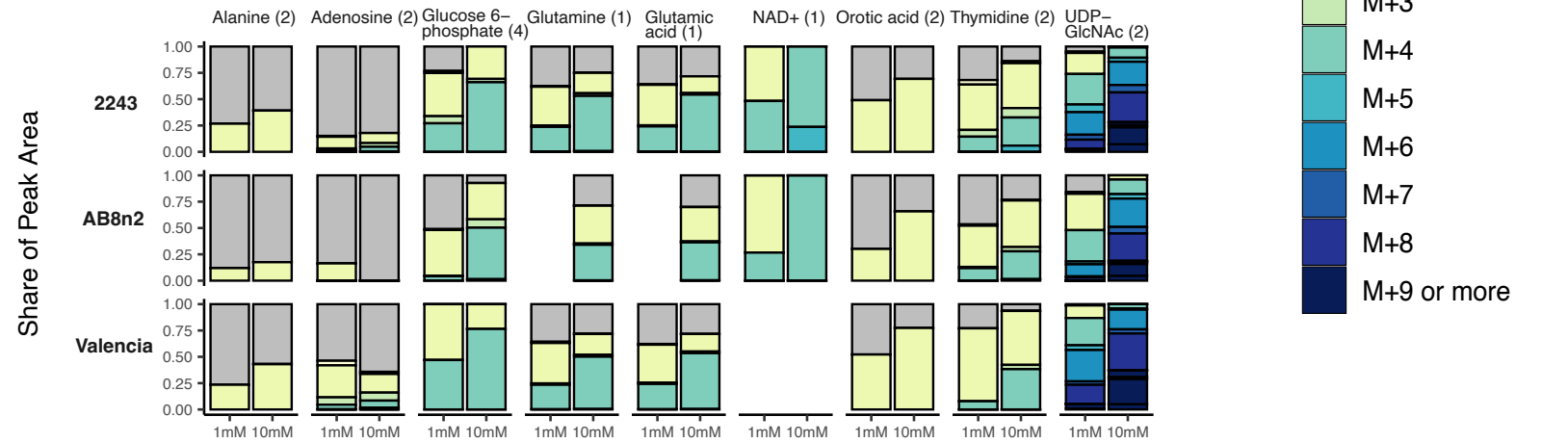

## D

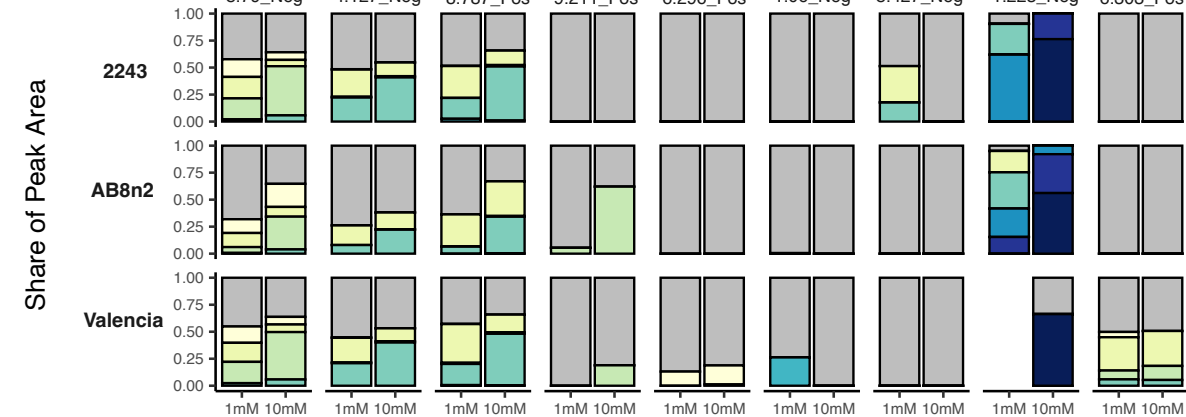

Supplement: S4 Fig — Related to Fig 2. (A) Growth of E. lenta strains in EDM1 with varying levels of sodium acetate (either stable isotope-labeled 13C2 or unlabeled). Optical density measurements were taken, and supernatant samples were collected at each indicated time point. Mean +/− standard error across 3 replicates is shown. (B) Average trajectories of labeled extracellular metabolites in 3 different strains of E. lenta. Metabolites shown are those with >50% and >5 × 10^4^ average peak area from labeled isotopologues in at least 1 time point in the 10 mM labeled acetate group. For metabolites detected in both positive and negative ionization mode, only positive mode is shown. The value in parentheses indicates the MSI annotation confidence level for each metabolite. (C) Labeled metabolites of known identity in intracellular extracts across 3 strains of E. lenta (data for DSM 2243 matches Fig 2E). Each panel shows the average mass isotopologue distribution across 3 replicates for a single metabolite in intracellular extracts from time point 5 (39 hours, late exponential phase). Metabolites are labeled with the compound name and MSI annotation confidence level in parentheses. Metabolites included are those with >15% and >104 average peak area from labeled isotopologues in either the 1 mM or 10 mM labeled acetate group. N-acetylated amino acids are excluded for space and reported in S1 Data. The isotopologue color legend is the same as in panel (B). (D) Labeled metabolites of unknown identity across 3 strains of E. lenta. Each panel shows the average mass isotopologue distribution (across 3 replicates) for a single metabolite in intracellular extracts from time point 5 (39 hours, late exponential phase). Metabolites are labeled with their estimated exact mass, retention time, and ionization mode. Metabolites included are those with >15% and >104 average peak area from labeled isotopologues in either the 1 mM or 10 mM labeled acetate group. The isotopologue color legend is the same as in [file pbio.3002125.s004.pdf]

**A**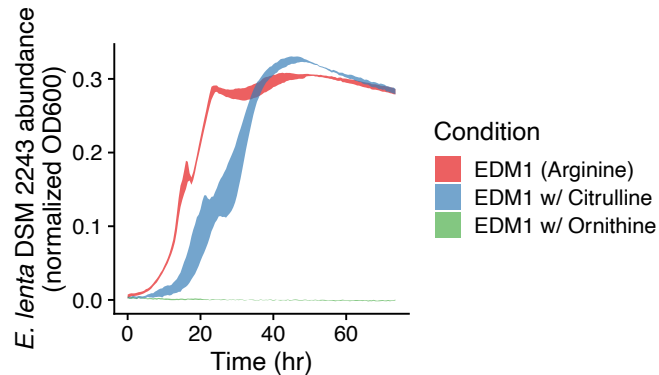**B**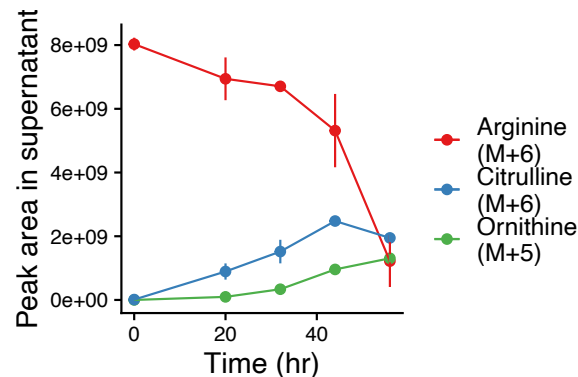**C****Extracellular metabolites**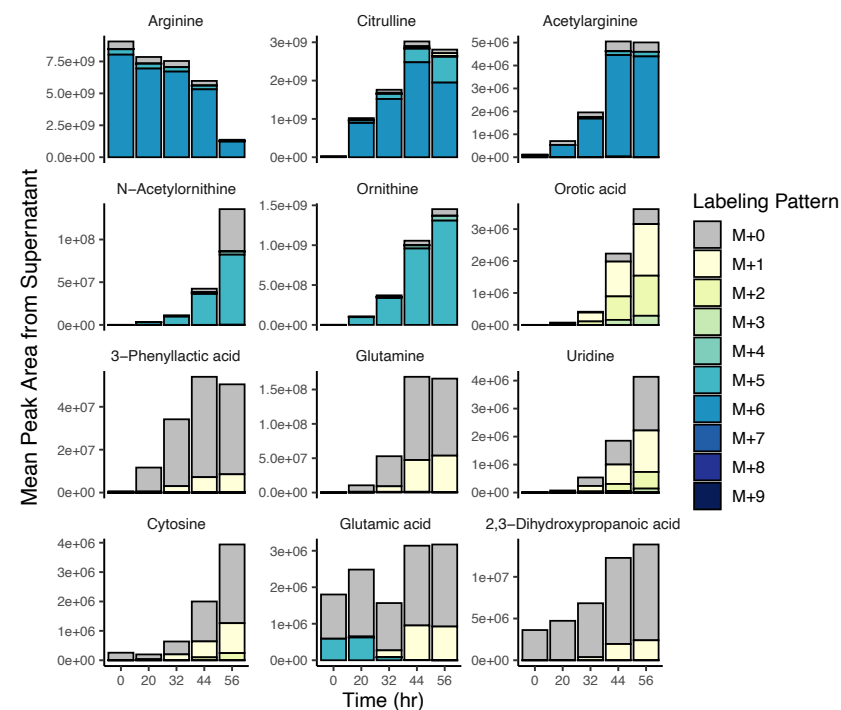**D****Intracellular metabolites**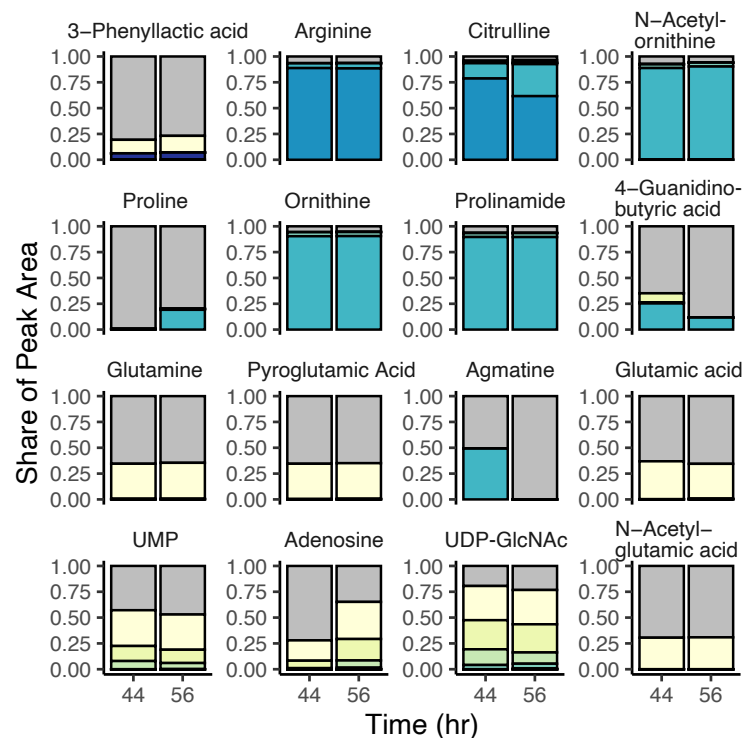**E**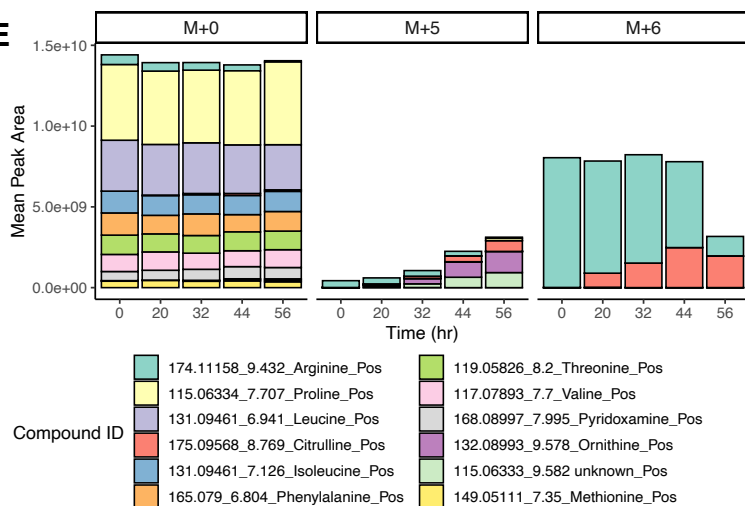**F**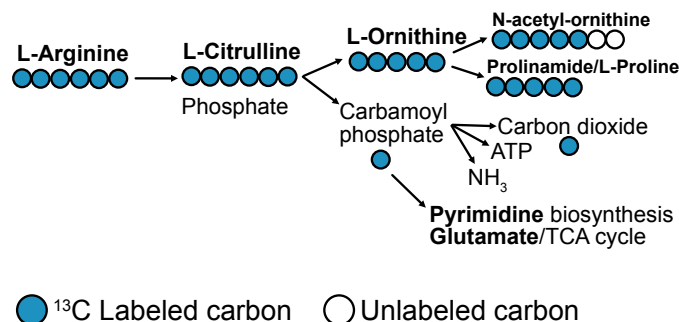

Supplement: S5 Fig — Related to Fig 2. (A) Citrulline, but not ornithine, has a similar effect as L-arginine on E. lenta growth. Growth curves of E. lenta grown in EDM1 media where the 1% L-arginine (red) has been replaced with an equimolar quantity of either L-citrulline (blue) or L-ornithine (green). Curves show mean +/− standard error across 4 replicates. (B) In E. lenta DSM 2243 cultures grown with 1% 13C6 labeled arginine, correspondingly labeled citrulline and ornithine accumulate in supernatants over the course of growth. Curves show mean +/− standard error across 3 replicates. (C) Mass isotopologue distributions of extracellular metabolites. Each barplot shows the isotopologue mean peak areas for each feature over time. Compounds shown are those of known identity that increase by a factor of at least 24, have at least 1 isotopologue with a peak area of greater 106 in at least 1 time point, and have a labeled isotopologue with >3% abundance in at least 1 time point. (D) Mass isotopologue distributions of intracellular metabolites. Each barplot shows the mean peak areas of isotopologues for each feature at 2 time points. Compounds shown are those of known identity with an average labeled MID > 0.1 and a total peak area from labeled isotopologues of at least 105 in at least 1 time point. The isotopologue color legend is the same as in panel (C). (E) Distribution of total signal of extracellular metabolites across labeling patterns. While signal from numerous unlabeled compounds is detected over time (left), compounds with M+5 labeling patterns are mainly restricted to ornithine, citrulline, and a compound of unknown identity (middle), and compounds found with high signal as M+6 isotopologues are mainly arginine and citrulline (right). Compounds shown are those with the highest peak areas at the final time point in positive ionization mode. (F) Hypothesized pathways for metabolism of L-arginine by E. lenta. Circles indicate the number of carbon atoms in selected compounds and are co [file pbio.3002125.s005.pdf]

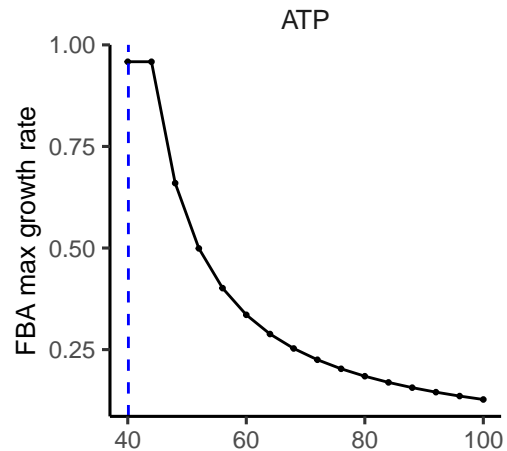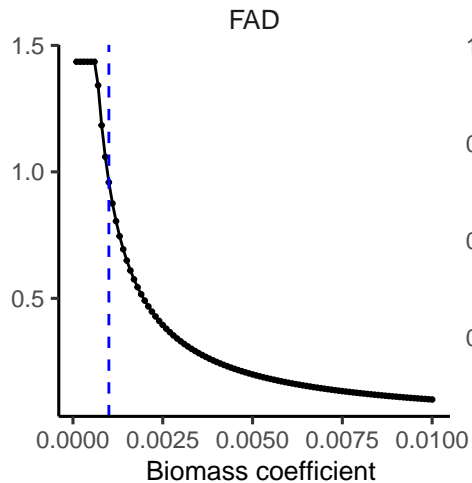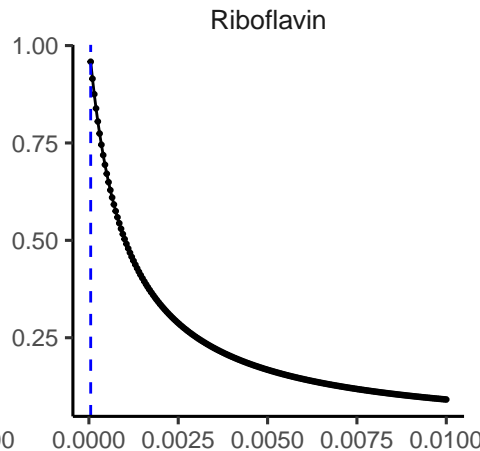

Value in iEL2243\_2

Supplement: S6 Fig — Related to Fig 3. Each panel shows the effect of varying the coefficient of the biomass equation for selected components on FBA-estimated maximum growth rate in EDM1 media. Compounds shown are those whose biomass coefficients have the largest effects on estimated growth rate. EDM1, Eggerthella Defined Media 1; FBA, flux balance analysis. (PDF) [file pbio.3002125.s006.pdf]

**A**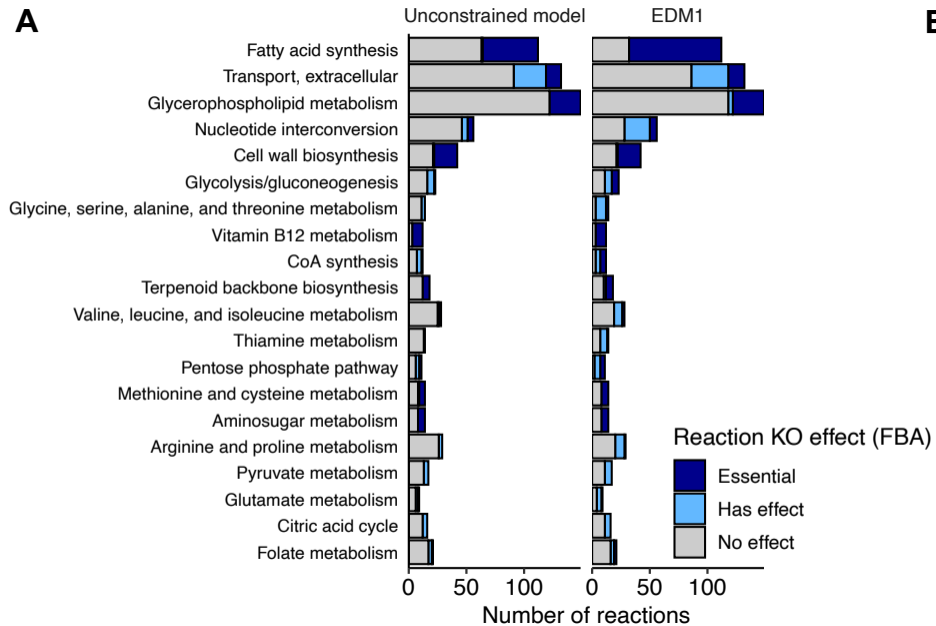**B**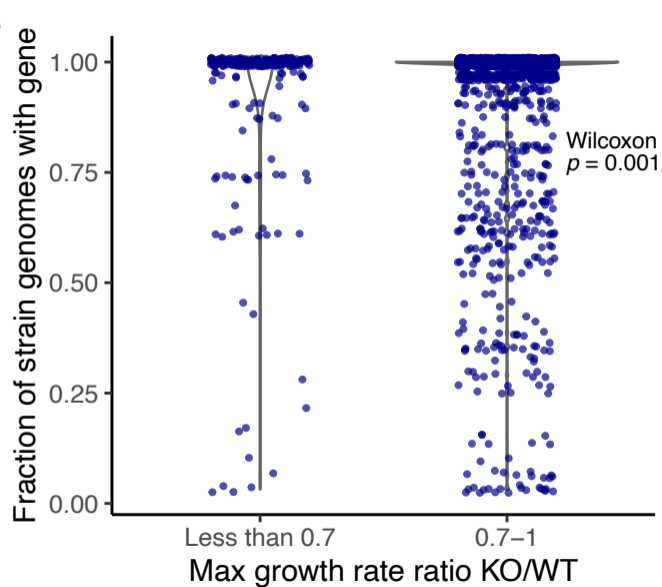

Supplement: S7 Fig — Related to Fig 3. (A) Predicted effects of knocking out reactions in the top 20 largest subsystems on growth of E. lenta, according to pFBA analysis of the iEL2243_2 model. Reactions designated “Has effect” are those for which the knockout has a predicted maximum growth rate less than wild-type but greater than 0. Essential reactions are those that reduced biomass flux to 0 when removed from the model. (B) Reactions linked to more conserved gene families are more likely to have substantial effects on growth when removed. Each point represents a reaction, separated on the x axis by whether the model without that reaction grew at >70% of the wild-type model. The y axis indicates the fraction of E. lenta strain genomes in which gene families (defined using ProteinOrtho clustering) linked to that reaction were present. (PDF) [file pbio.3002125.s007.pdf]

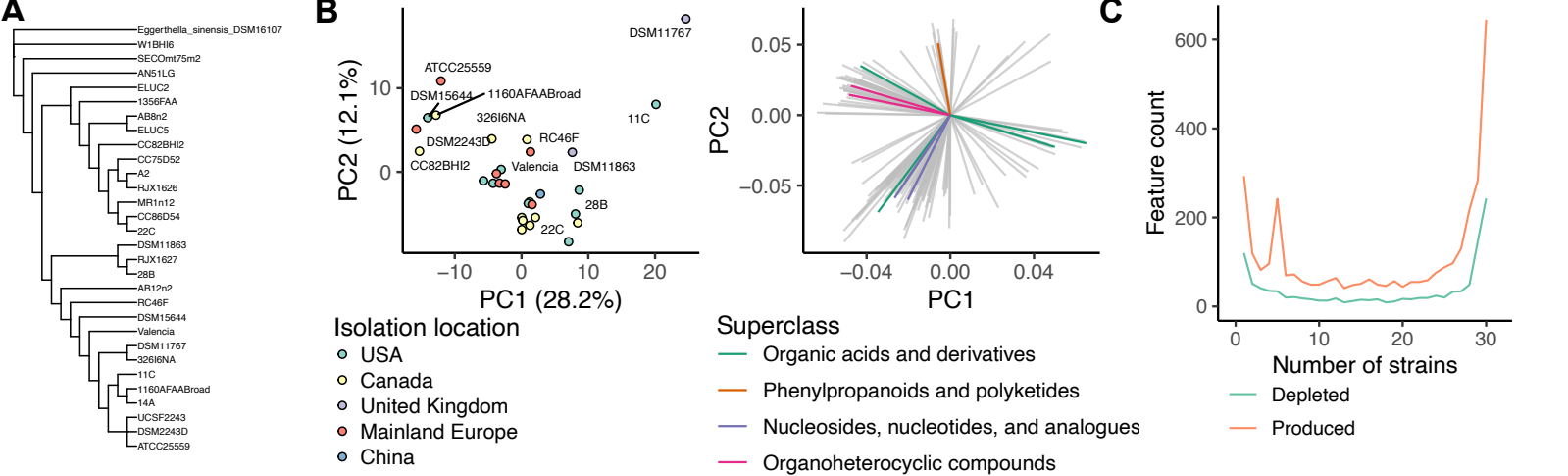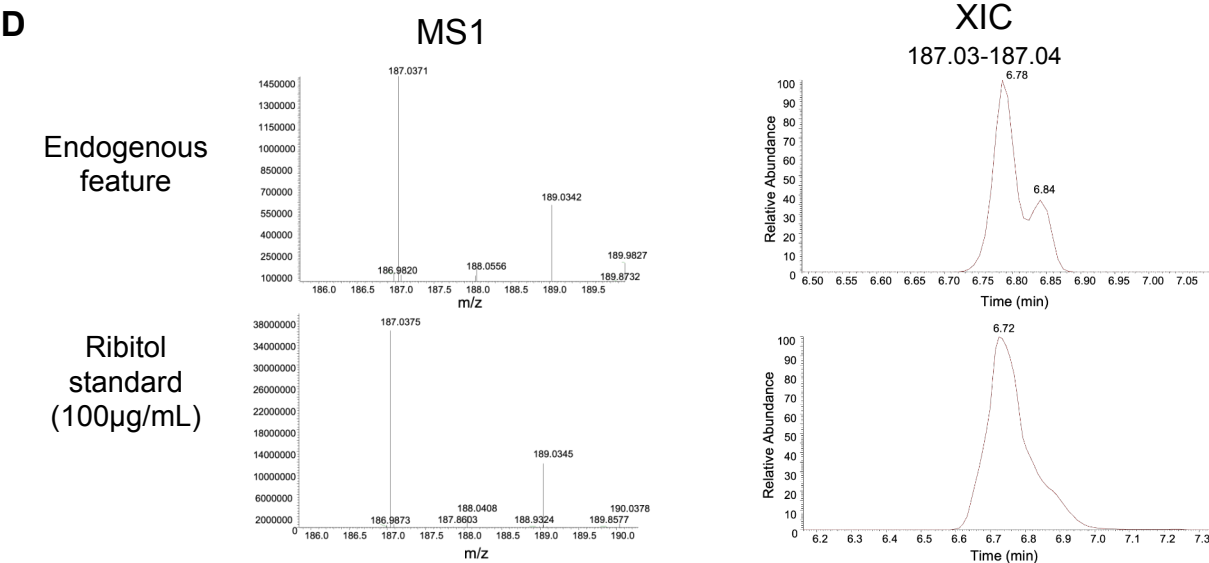

Supplement: S8 Fig — Related to Fig 4. (A) Phylogeny of 30 Eggerthella strains analyzed in this study. This phylogeny was previously constructed based on core gene alignments using Phylophlan [26]. (B) PCA of log-transformed metabolite intensity profiles of stationary phase supernatants from 30 Eggerthella isolates in EDM1. In the left-hand panel, color indicates the geographic origin of each isolate. The right panel shows the largest feature loadings for the PCA and their corresponding chemical classes as assigned by GNPS, where available. Dereplicated metabolite features with an average value > 105 in at least 1 strain were included. (C) Distribution of the number of strains producing or depleting each metabolite feature. Features included are those that were significantly modified by at least 1 Eggerthella isolate in this experiment (FDR-adjusted p-value < 0.1 and log2 fold change > 0.5). (D) Comparison of MS1 spectra and extracted ion chromatograms for the unidentified features shown in Fig 4D in a representative sample, compared with a pure ribitol standard spiked into sterile EDM1 at a concentration of 100 μg/mL. The data shown in the top row are from the biological sample, while the data in the bottom row are from the ribitol standard. (E) Map of the teichoic acid biosynthesis region of the genome of representative Eggerthella strains. Genes outlined in bold are the gene families associated with the unidentified metabolite features shown in Fig 4D. Gene regions were defined in each genome based on the location of the genes annotated as tagG and tagH by Prokka. EDM1, Eggerthella Defined Media 1; FDR, false discovery rate; PCA, principal component analysis. (PDF) [file pbio.3002125.s008.pdf]

**A**

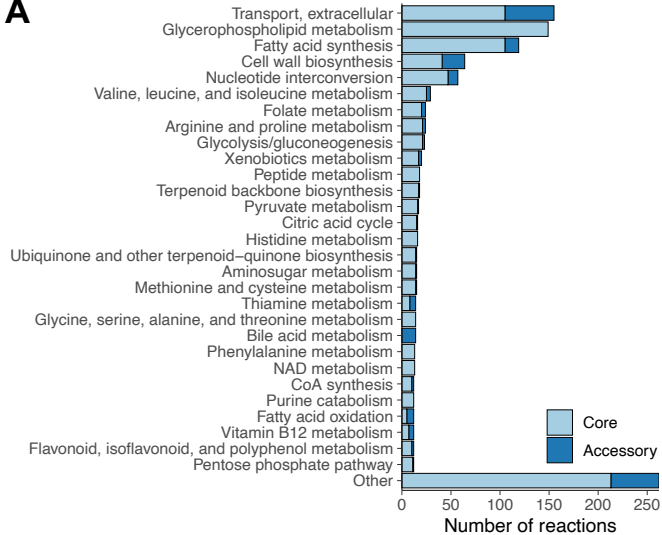

# B

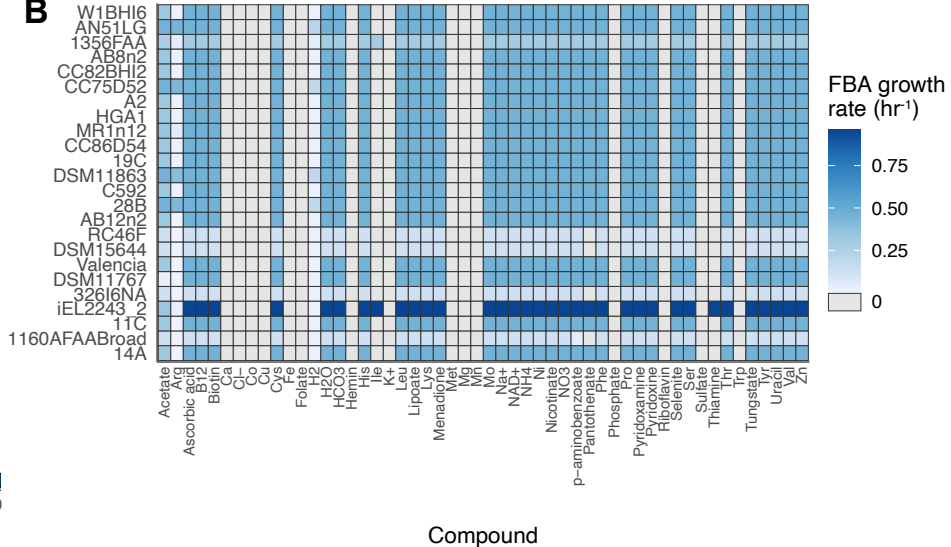

Supplement: S9 Fig — (A) Distribution of core and accessory reactions across subsystems, based on comparative analysis of metabolic reconstructions of 24 E. lenta strain genomes. (B) Predicted maximum growth rate inferred by flux balance analysis of each of the 24 E. lenta strain reconstructions in 52 leave-one-out media conditions based on EDM1. Gray tiles indicate predicted cases of zero growth. (PDF) [file pbio.3002125.s009.pdf]

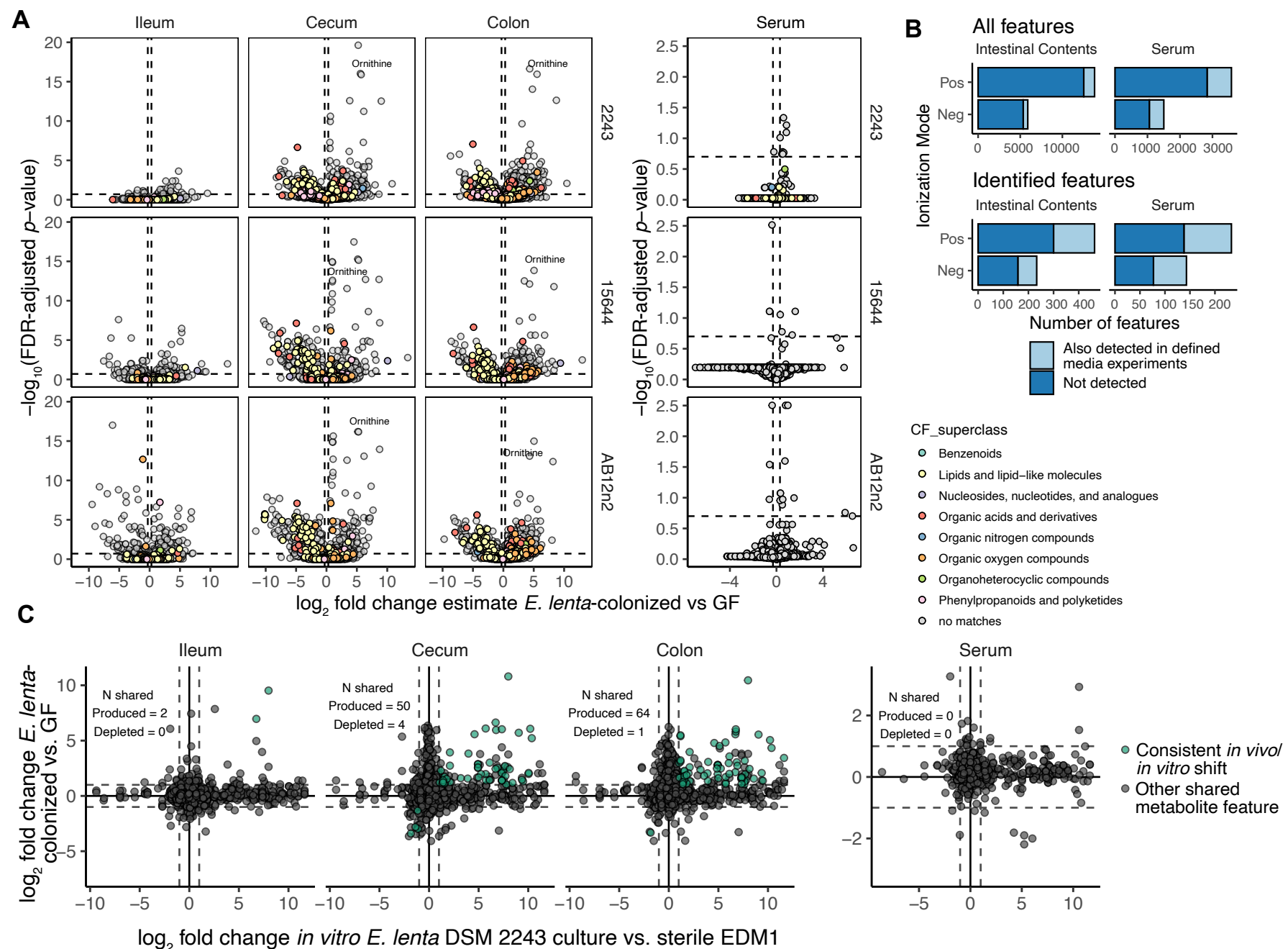

Supplement: S10 Fig — Related to Fig 5. (A) Volcano plots of differential abundance analysis of metabolite features in intestinal contents and serum of gnotobiotic mice monocolonized with one of 3 E. lenta strains. Effect sizes and significance are estimated from group comparisons based on linear mixed models of log-transformed metabolite abundances, accounting for animal and cage random effects. (B) Total number of untargeted metabolomics features in intestinal contents and serum of gnotobiotic mice that could be linked to features in either of 2 in vitro EDM1 metabolomics datasets, based on high similarity of m/z, retention time, and MS2 spectra. (C) Comparison of the effect of E. lenta DSM 2243 on metabolites detected in both EDM1 cultures in the untargeted time course experiment and monocolonized mice. Each point represents a metabolite feature detected in both datasets. The x axis indicates the log2 fold change of each feature in supernatants from the E. lenta DSM 2243 time course experiment compared with sterile controls, compared with the covariate-adjusted log2 fold change of that feature in monocolonized mice compared with GF mice. Points are colored green if the feature is significantly differentially abundant in gnotobiotic mice and is shifted in the same direction by the corresponding strain in the time course in vitro experiment. EDM1, Eggerthella Defined Media 1; GF, germ-free. (PDF) [file pbio.3002125.s010.pdf]

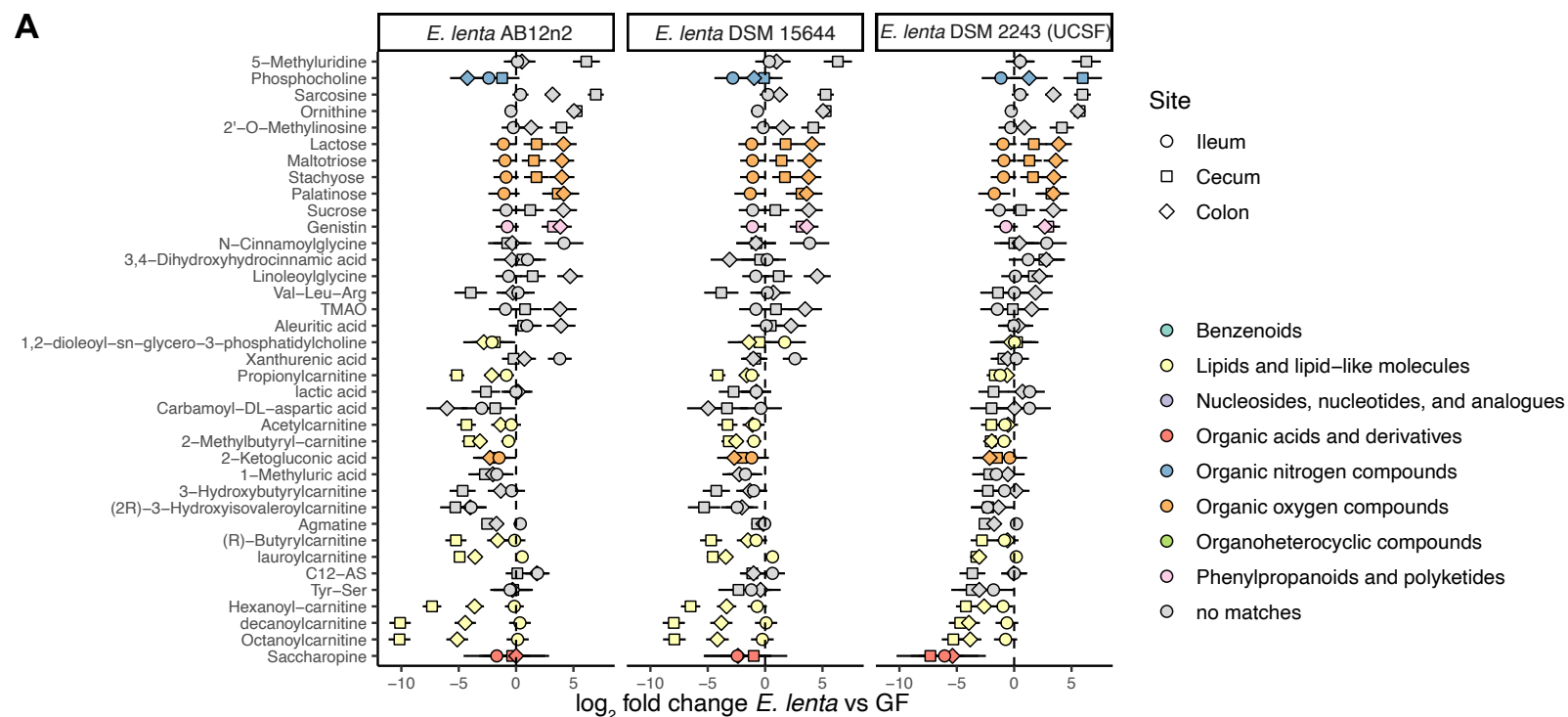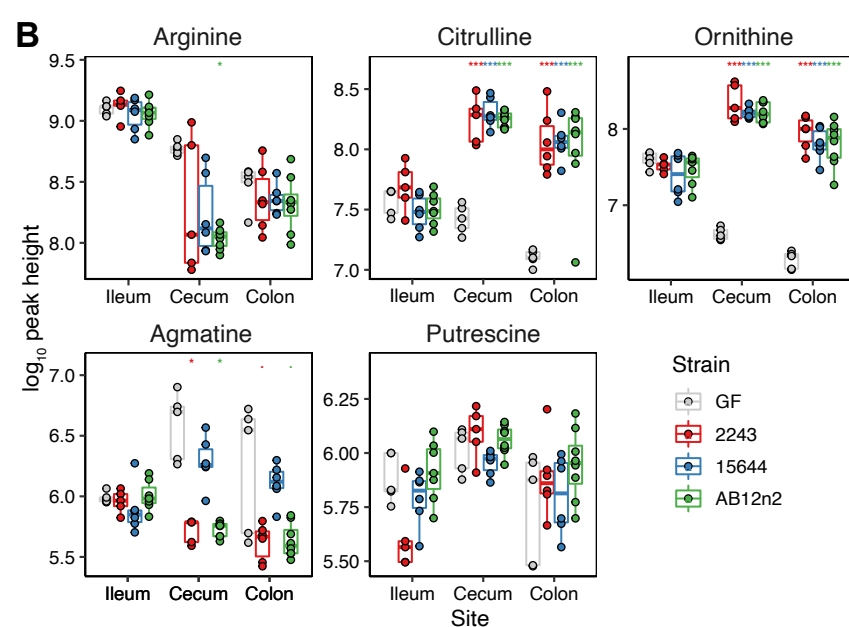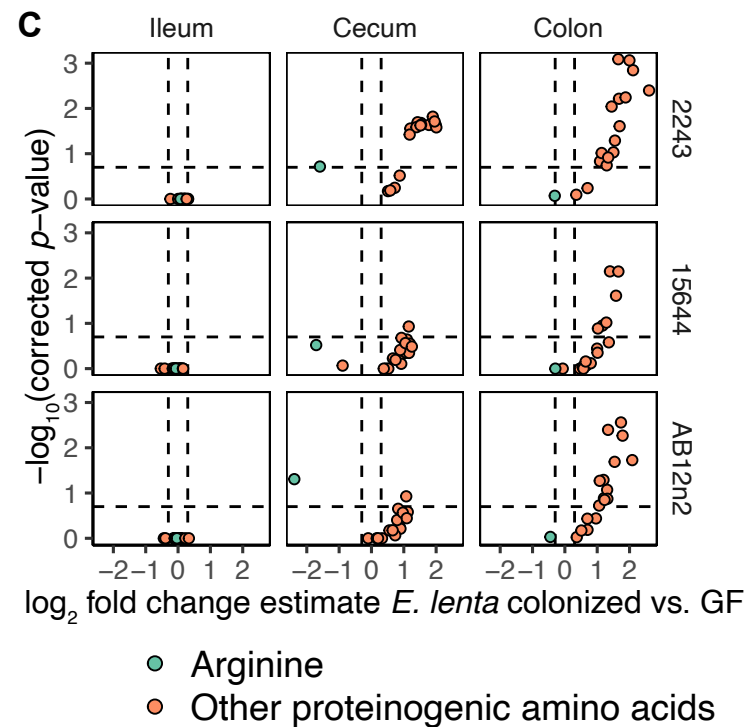

Supplement: S11 Fig — Related to Fig 6. (A) Annotated metabolites with the largest shifts in intestinal contents of E. lenta-colonized mice compared with GF. Metabolites are shown if they were identified based on library comparison and were among the 600 most strongly shifted features in any individual site or colonization group, based on linear mixed models. Each point shows the effect size in a single site, and color indicates chemical class where available (assigned using feature-based molecular networking with GNPS). (B) Abundance of arginine and agmatine-related metabolites in gnotobiotic mice. Arginine is only slightly depleted by E. lenta, although its expected products, ornithine and citrulline, are greatly increased. Agmatine is significantly depleted, while its expected product, putrescine, is not significantly increased. “.” indicates Benjamini–Hochberg adjusted p < 0.1, *p < 0.05, **p < 0.01, ***p < 0.001. (C) Volcano plots illustrating shifts in the abundance of proteinogenic amino acids in E. lenta-colonized mice. Arginine is colored in green. Effect sizes and significance are estimated from group comparisons based on linear mixed models of log-transformed metabolite abundances, accounting for animal and cage random effects. (PDF) [file pbio.3002125.s011.pdf]

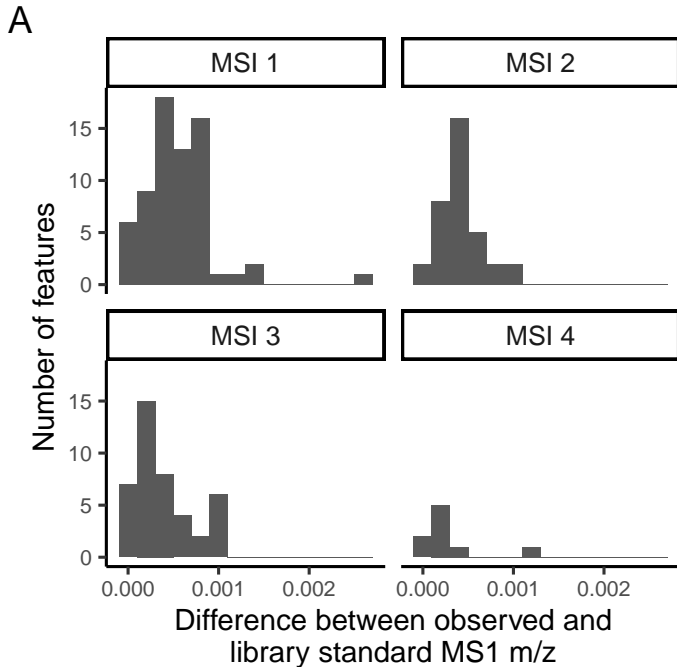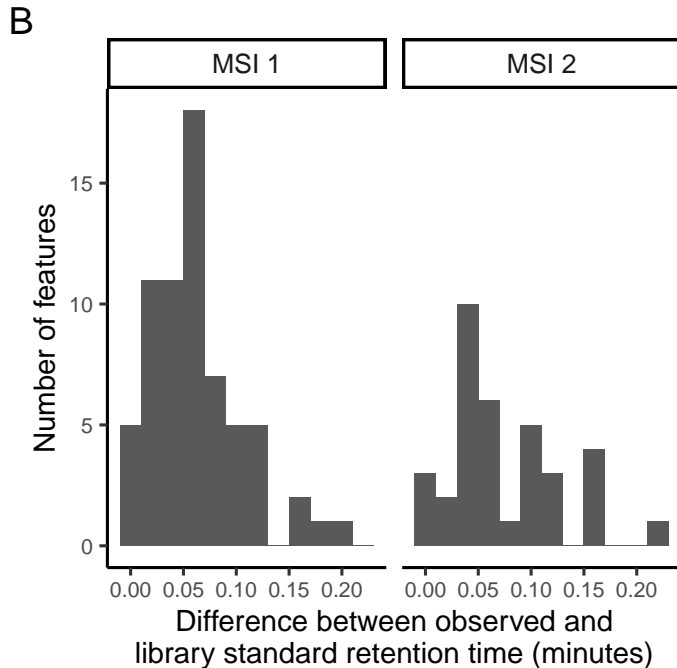

Supplement: S12 Fig — Related to Materials and methods. (A) Difference between library standard m/z and average observed m/z for annotated metabolite features in the EDM1 time course dataset, separated by MSI annotation confidence level. (B) Difference between library standard retention time and average observed retention time for annotated metabolite features in the EDM1 time course dataset, separated by MSI annotation confidence level. Features assigned MSI of 3 or 4 did not necessarily have retention times matched with a library standard. EDM1, Eggerthella Defined Media 1; MSI, Metabolomics Standards Initiative. (PDF) [file pbio.3002125.s012.pdf]
